# Successful management of a rare case of juvenile giant right ventricular myxoma

**DOI:** 10.3389/fsurg.2022.1102742

**Published:** 2023-01-11

**Authors:** Ke Gong, Yifeng Yang, Yadan Shen, Haidan Liu, Li Xie, Jijia Liu

**Affiliations:** ^1^ Department of Cardiovascular Surgery, The Second Xiangya Hospital of Central South University, Central South University, Changsha, China; ^2^ Extracorporeal Life Support Center of Cardiovascular Surgery, The Second Xiangya Hospital of Central South University, Central South University, Changsha, China

**Keywords:** primary cardiac tumors, right ventricle, diagnosis, treatment, surgery

## Abstract

Primary cardiac tumors are extremely uncommon in young children and infants. Cardiac myxoma are typically found in the atria, predominately in the left atrium, with relatively few found on the right side, such as in the right ventricle or pulmonary artery. Numerous significant complications, including sudden death, can result from obstruction of the main pulmonary artery trunk and right ventricular outflow tract. Here, we describe the case of a 14-year-old Chinese girl diagnosed with a right ventricular myxoma located in the right ventricle and extended into the main pulmonary trunk. Complete resection of the myxoma and histological confirmation were performed.

## Introduction

Myxomas occur in all age groups, most commonly between the ages of 30 and 60, and are predominantly found in females. Rhabdomyomas and teratomas are the most common tumors found in children, while myxomas and fibroids are less common. They are most commonly found in the left atrium. The right atrium (RA) is the second most frequent place where myxomas arise, constituting about 7%–12% of cases. Only a few cases of myxoma found in the right ventricle have been documented (1–4). The majority of medical professionals advise early surgical resection to lower mortality brought on by complications, such obstruction of the heart's inflow or outflow system (5). Here, we present a case of a primary cardiac myxoma in a child that had protruded into the pulmonary trunk from the right ventricle (RV).

## Case report

This study obtained the informed consent of the patients and their families for publication. Due to chest congestion and edema, a 14-year-old girl with nephrotic syndrome who had been identified in a smaller district hospital in China was sent to our division. Her blood pressure was 109/80 mmHg at the time of admission, and her pulse rate was 107 beats per minute. Physical examination revealed face and ankle edema as well as a grade 4/6 systolic ejection murmur at the left upper sternal border.

**Figure 1:**
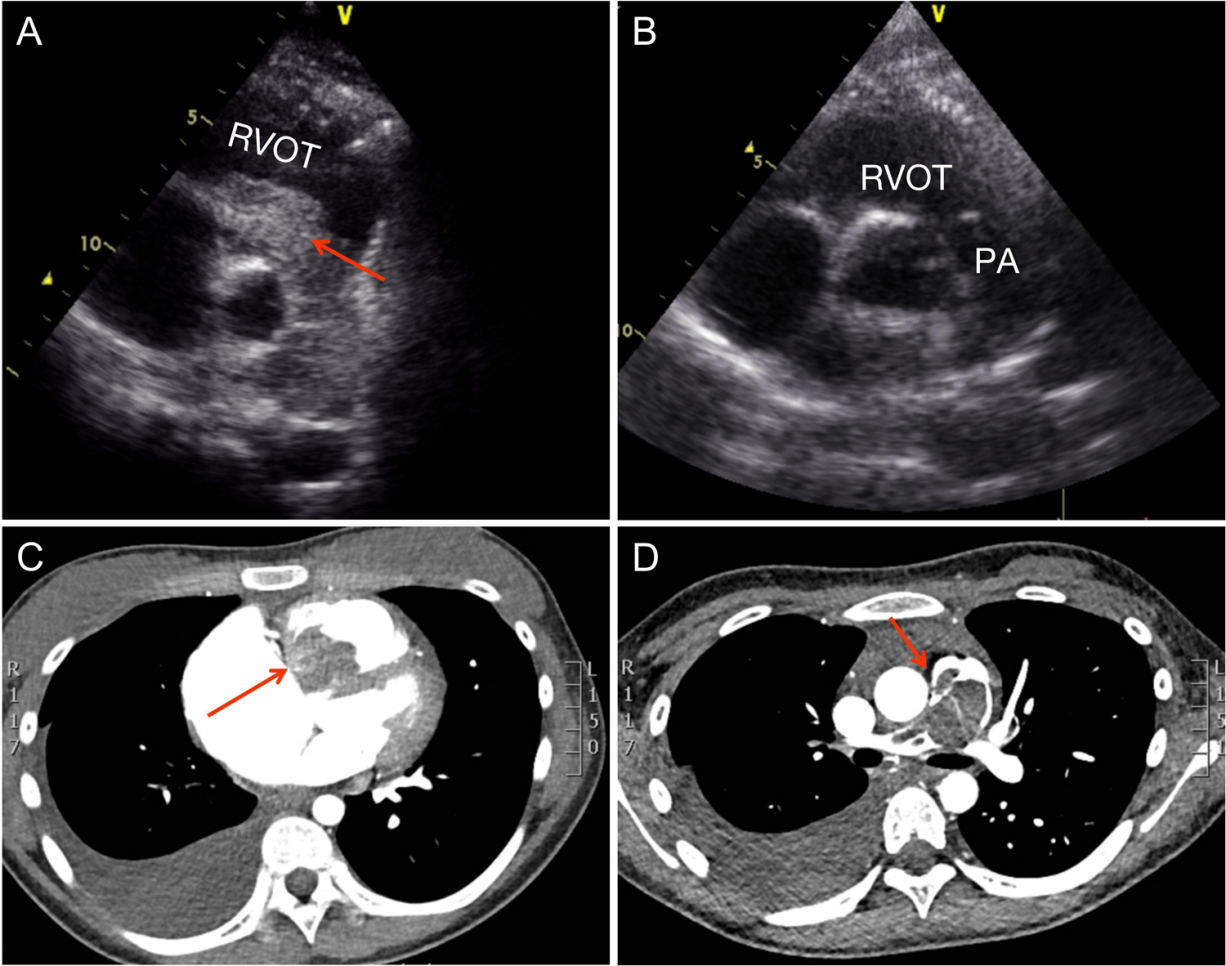
Preoperative and postoperative examination. (**A**) Preoperative parasternal short-axis view at the level of the aortic valve showing the obstructed right ventricular outflow tract and pulmonary trunk. (**B**) Postoperative parasternal short-axis view at the level of the aortic valve showing the unobstructed right ventricular outflow tract and pulmonary trunk. (**C**) Computed tomography reveals the right ventricular outflow tract. (**D**) Computed tomography reveals the pulmonary trunk and right pulmonary artery. RVOT, right ventricular outflow tract; PA, pulmonary artery.

Transthoracic echocardiography revealed a significant 70 × 30 mm RV mass which protruded into the right ventricular outflow tract (RVOT) and the pulmonary trunk. The pressure gradient from the pulmonary artery to the RV was 64 mmHg (Figure 1A). There was also enlargement of the RV and right axis deviation along with sinus tachycardia. A filling deficit in the RV, pulmonary trunk, and right pulmonary artery was discovered by computed tomography (CT) (Figures 1C,D).

**Figure 2:**
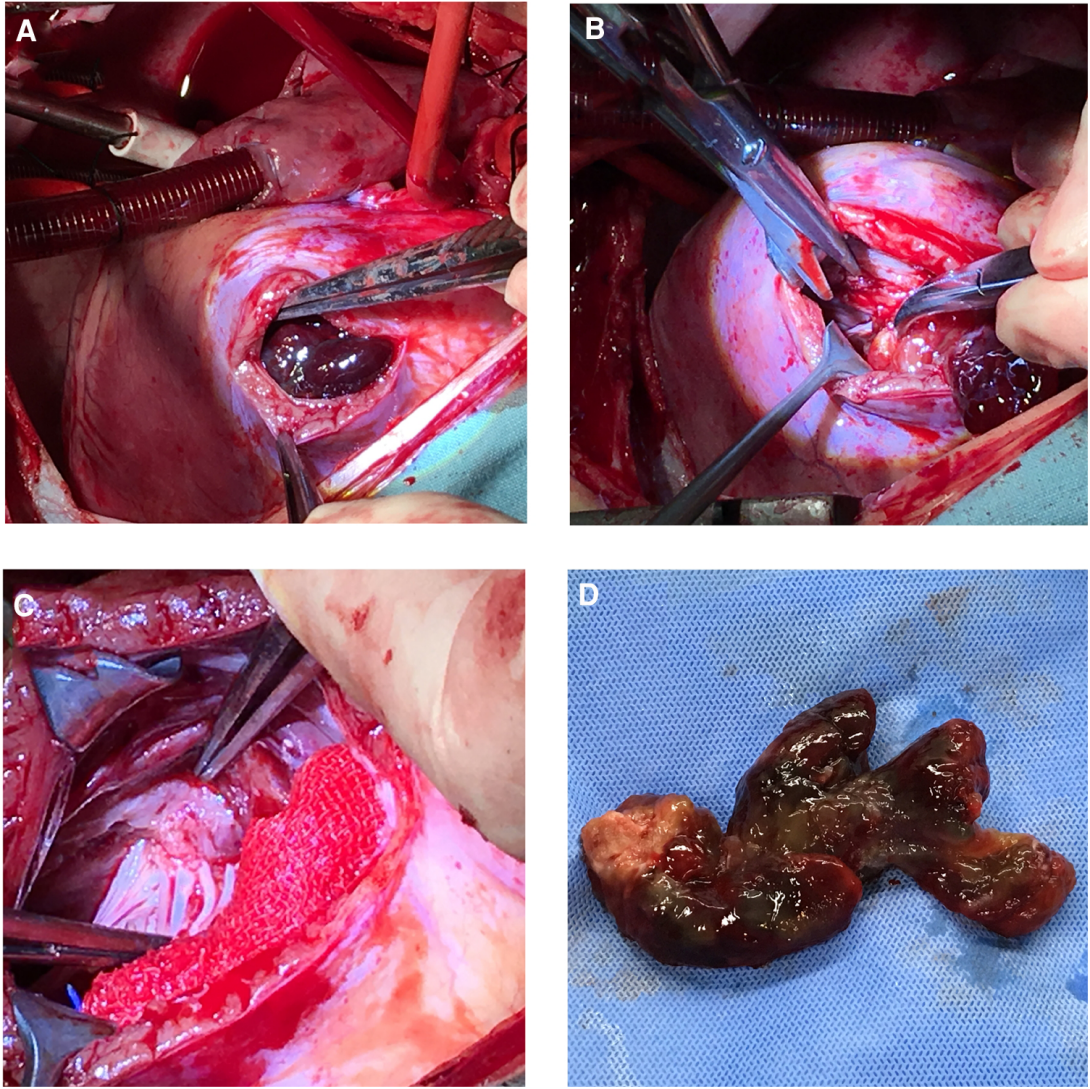
Surgery pictures. Operative procedures. (**A**) Right ventricular outflow tract incision was made to expose the tumor. (**B**) and (**C**) The tumor root which attached to the membranous portion of interventricular septum was surgically removed. (**D**) The entire tumor.

She underwent a sternotomy and cardiopulmonary bypass creation by aortic and bicaval cannulation under general anesthesia. She had her RA and RVOT opened. The myxoma was attached to the membranous portion of interventricular septum and had grown into the main pulmonary artery and the right pulmonary artery. The mass was entirely eliminated (Figure 2). According to pathological findings, there were two pieces of tumor tissue totaling 5 × 4 × 2 cm, the majority of which was mucus. It appeared to be a myxoma under a microscope, with slightly larger individual nuclei. CD34(vascular+), NSE(−), SMA(+), CK(−), CD68(+), MC(−), CR(+), Desmin(−), CEA(−), EMA (1), and F8(+) were immunohistochemically positive. She was discharged 7 days after surgery and the recovery time was uncomplicated. Following surgery, echocardiography showed an unobstructed RVOT (Figure 1B). Seven-year postoperative follow-up showed no recurrence of the patient's tumor. The entire process of disease development is described in Table 1.

**Table 1:** Timeline. The process of the patient's disease progression and treatment.

Timeline	3 months before hospitalization	14 days before hospitalization	3 days before hospitalization	Hospitalization	Day 2 of hospitalization	Day 4 of hospitalization	5:00 am on the day 5 of hospitalization	9:00 am on the day 5 of hospitalization	Day 11 after surgery	Follow-up review
Process	Finding symptoms	Hospitalization in other hospitals, diagnosis of nephrotic syndrome	Hyperechoic findings on heart ultrasound at other hospitals, pending confirmation	Transferred to our hospital pediatric department, blood test	Echocardiography	CT	Transferred to our cardiovascular surgery department	Surgery	Discharged from hospital	Annually

## Discussion

Up to 75% of myxomas are found in the left atrium. Right-sided myxomas are uncommon (15%–20%), and RV (3%–4%) or pulmonary artery myxomas are exceedingly uncommon (6). Syncope, pulmonary embolism, chest congestion, and sudden death are complications that can result from obstruction of the outflow of blood from the RV (2, 7).

Myxomas are uncommon in children despite being the most frequent primary heart tumor. A study reported that 8 children with a clinical diagnosis of cardiac tumor underwent surgery between 1986 and 2003. Surgical pathology only revealed myxomas in 2 patients (25%) (8). In another study, 56 children had primary heart tumors. Of those, 6 children had fibrosarcoma and 44 children had rhabdomyosarcoma. No myxomas were discovered (9). Thus, pediatric cardiac myxomas are quite uncommon. Although right ventricular myxomas in children have been documented in the past, pediatric cases of enormous right ventricular mucinous tumors that extend into the pulmonary artery are extremely rare. Patients of this type are at a significant risk for sudden death and pulmonary embolism.

It is especially useful for the diagnosis and treatment of patients to properly represent the size, quantity, and attachments of tumors. Consequently, it is crucial to appropriately describe cardiac tumor using imaging. CT, MRI and echocardiography are frequently used to assess cardiac tumor, according to the guidelines. The location, size, form, attachment points, and motion features of mucinous tumors can be determined *via* transthoracic echocardiography and, if required, transesophageal access. CT provides a better assessment of tumor extent, including invasion of adjacent vessels and pulmonary metastases, than echocardiography. In addition, CT is capable of directly imaging tumorigenic pulmonary emboli and can also be used to assess calcification (10). MRI can assess myocardial infiltration, pericardial involvement, and/or extracardiac extension. MRI overcomes the usual limitations of echocardiography and can more accurately assess changes in cardiac function. The use of intravenous contrast agents has improved tumor characterization and delineation of tumor boundaries. MRI can also differentiate tumors from other non-tumor masses (11–13). In our case, the patient did not undergo the MRI, because MRI is still a relatively expensive examination. The financial burden on the patient is why this examination was not performed. Although not used in our report, MRI is still important as an adjunct to diagnose tumors and determine the type of tumor.

In our case, the symptoms of chest tightness, facial edema, and lower extremity edema were so first diagnosed as nephrotic syndrome. This may be due to a possible lack of cardiac auscultation. Therefore, in suspicious cases, a brief auscultation and routine imaging should be performed at the regional hospital before the patient is referred to a higher level hospital. This is important because delays in diagnosis and treatment can lead to complications and, in severe cases, death.

Since no medications have been developed to diminish or stop the growth of myxomas, surgical excision is the only effective treatment for them in any heart cavity. The surgical plan includes complete removal of the tumor while restoring any tumor-affected valves that may be present. Vigorous palpation and other cardiac manipulation should be avoided until extracorporeal circulation is started since the threat of tumor fragmentation and embolization remains high. Asymptomatic myxoma patients have also undergone surgery with positive outcomes and no postoperative fatalities (14, 15). Recurrence has been observed in approximately 5% of patients months or years after surgery. On the surgical approach, some surgeons choose either the RA or RVOT approach. We chose both the RA and RVOT approach because the root of the patient's tumor was located in the membranous portion of interventricular septum and entered the pulmonary artery along the RVOT. Therefore, we believe that a simple right atrial incision is not sufficient to remove the tumor cleanly, so we choose a dual approach for an incision. After the operation, the child did very well and was almost back to normal upon discharge from the hospital. Rarely, a RV myxoma will block the RVOT in children (16). However, immediate and thorough surgical resection should be performed to prevent outflow tract occlusion, which could cause rapid mortality.

## Data Availability

The original contributions presented in the study are included in the article/Supplementary Material, further inquiries can be directed to the corresponding author/s.
